# Contrast-Enhanced Sonography of the Liver: How to Avoid Artifacts

**DOI:** 10.3390/diagnostics14161817

**Published:** 2024-08-20

**Authors:** Hiroko Naganuma, Hideaki Ishida, Hiroshi Nagai, Atushi Uno

**Affiliations:** 1 Department of Gastroenterology, Yokote Municipal Hospital, Yokote 013-8602, Japan; 2 Department of Gastroenterology, Akita Red Cross Hospital, Akita 010-1495, Japan; minnnanous@gmail.com; 3 New Generation Imaging Laboratory, Tokyo 168-0065, Japan; h-nagai@jcom.home.ne.jp; 4 Department of Gastroenterology, Ohmori Municipal Hospital, Yokote 013-0525, Japan; ash_uno@yahoo.co.jp

**Keywords:** contrast-enhanced sonography, liver, artifact avoidance, image misinterpretation, diagnostic pitfall

## Abstract

Contrast-enhanced sonography (CEUS) is a very important diagnostic imaging tool in clinical settings. However, it is associated with possible artifacts, such as B-mode US-related artifacts. Sufficient knowledge of US physics and these artifacts is indispensable to avoid the misinterpretation of CEUS images. This review aims to explain the basic physics of CEUS and the associated artifacts and to provide some examples to avoid them. This review includes problems related to the frame rate, scanning modes, and various artifacts encountered in daily CEUS examinations. Artifacts in CEUS can be divided into two groups: (1) B-mode US-related artifacts, which form the background of the CEUS image, and (2) artifacts that are specifically related to the CEUS method. The former includes refraction, reflection, reverberation (multiple reflections), attenuation, mirror image, and range-ambiguity artifacts. In the former case, the knowledge of B-mode US is sufficient to read the displayed artifactual image. Thus, in this group, the most useful artifact avoidance strategy is to use the reference B-mode image, which allows for a simultaneous comparison between the CEUS and B-mode images. In the latter case, CEUS-specific artifacts include microbubble destruction artifacts, prolonged heterogeneous accumulation artifacts, and CEUS-related posterior echo enhancement; these require an understanding of the mechanism of their appearance in CEUS images for correct image interpretation. Thus, in this group, the most useful artifact avoidance strategy is to confirm the phenomenon’s instability by changing the examination conditions, including the frequency, depth, and other parameters.

## 1. Introduction

Contrast-enhanced sonography (CEUS) is the most important and sensitive diagnostic imaging tool in clinical settings [1,2]. It has been successfully applied to visualize a wide range of organs [3,4,5,6,7,8], but the most important target organ is the liver [9,10]. CT, MRI, and CEUS each have their own merits. Although CEUS has some limitations, such as operator dependency and a lack of strictly established machine standardization, its advantages include its renal non-toxicity, cost efficacy, and excellent temporal and spatial resolutions, offering dynamic imaging of more than 10 real-time images per second [8,10,11,12]. This dynamic imaging permits us to observe hemodynamic changes in the liver [12]. The most important diagnostic points of CEUS for liver tumors are (1) the onset time, the mode of wash-in in the early arterial phase, and the mode of late wash-out [2,13,14,15,16]. Combining these CEUS findings allows for the characterization of a wide spectrum of benign and malignant focal liver lesions [11,12]. However, as its use has become widespread, CEUS is semi-routinely performed by less experienced personnel without scientific foundations. Ultrasound (US) images, including CEUS images, are constructed purely via US physics [17]. With the continuous improvement of US technology, CEUS has gained new applications, including three-dimensional display [18], parametric display of quantitative contrast information ^1)^ [19], multi-parametricity [20], intraoperative use [21], endoscopic procedure guidance [22], robot-assisted examination [23], AI-assisted analysis [24], fusion imaging [25], remote diagnosis [26], and combined use with microflow imaging ^2)^ [27]. However, despite these many new US technologies, there still remains a paucity of data on CEUS artifacts. US technology is always associated with possible artifacts, and sufficient knowledge of both US physics and these artifacts is indispensable to avoid the misinterpretation of all kinds of US images [28,29]. For CEUS, the diagnostic difficulty is thought to be much deeper than that for conventional US because of the many complex parameters. This important diagnostic problem motivated us to classify the CEUS artifacts encountered in daily CEUS examinations and analyze their possible mechanisms. To provide this background, this review aims to explain the basic physics of CEUS, classify the associated artifacts, and provide some examples of how to avoid them. In this review, we hope to provide the latest knowledge on CEUS to promote its appropriate use in the liver in daily clinical settings.

^1)^ Parametric quantitative display of contrast US data: A quantitative analysis of a selected region of interest (ROI) can be measured, and the average contrast signal intensity can be calculated. This signal intensity within the ROI can be displayed as a function of time in the form of a time–intensity curve, which describes the wash-in and wash-out of the contrast medium in that ROI [19].

^2)^ Microvascular flow imaging: This new US technology involves the use of filters that are different from those of Doppler imaging. It reduces artifacts from tissue motion artifacts and increases the sensitivity to lower-velocity blood flow detection.

## 2. CEUS: Frame Rate, Image Quality, and Mode Selection

**Figure 1:**
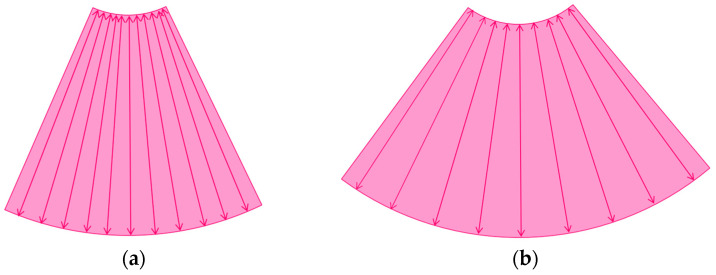

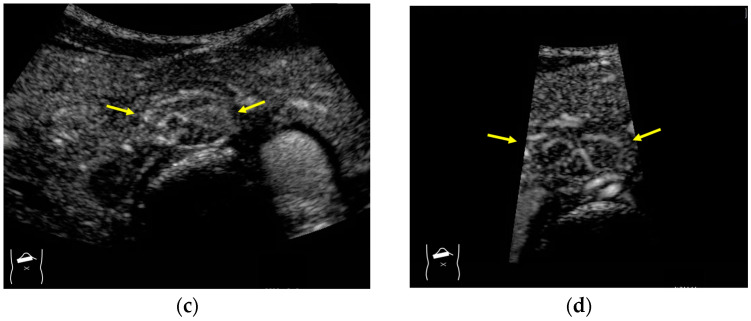
A schematic drawing of high-frame-rate CEUS: (**a**) narrowing the viewing angle; (**b**) decreasing the number of ultrasound beams; (**c**) representative CEUS image of the method (**a**,**c**): frame rate 12/s; (**d**): frame rate 30/s (focal nodular hyperplasia, arrows). A more detailed vascular structure (in this case, it has a spoke-wheel appearance) can be displayed using high-frame-rate CEUS when observing hypervascular mass lesions.

The image frame rate (the number of times/second) is a characteristic of US examinations, and it is closely related to the temporal resolution. The higher the frame rate, the faster the image movement [30,31], which allows for rapid survey scans of the target lesion [32]. Meanwhile, the number of US beam lines is generally related to the spatial resolution. The smaller the number of US beams, the faster the scanning speed. However, this can compromise the US image quality (Figure 1a,b). Thus, it is important to balance the frame rate and the image quality in US examinations. Another way to increase the frame rate is to reduce the image width, i.e., narrowing the view angle. This method can be easily performed even with middle-price CEUS machines (Figure 1c). Thus, there are two ways to gain high-frame rate CEUS images using current CEUS machines: (a) narrowing the frame angle (as shown in Figure 1c) and (b) decreasing the number of US beams. The former keeps the same spatial resolution and is suitable for observing small lesions. The latter is only available with high-end CEUS machines and is suitable for observing relatively large lesions.

The current high-end machines have the above two functions. By selecting function (b), the machine automatically decreases the number of US beams (usually to half of the usual number) and increases the frame rate (usually to twice the usual rate) without changing the width of the field of view. This function is especially useful when observing the vascular structures of high-flow liver tumors.

**Figure 2:**
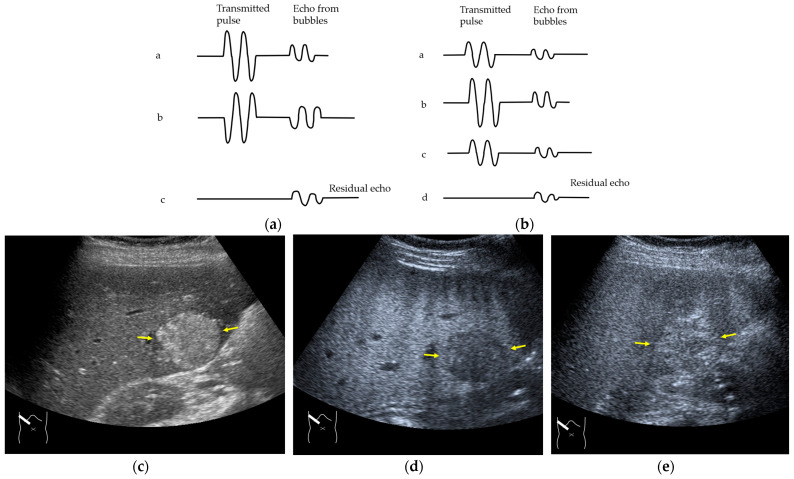
Amplitude modulation vs. the pulse inversion method: (**a**) Schematic drawing of pulse inversion (PI) mode. Substraction a − b = residual echo. (**b**) Schematic drawing of amplitude modulation (AM) mode. Substraction (a + c) − b = residual echo. (**c**) Gray-scale US shows a 4 × 4 cm heterogeneous mass (metastasis from colon cancer) (arrows) in the segment 6 (**c**). CEUS in amplitude modulation mode clearly demonstrates a punched out defect, leading to the diagnosis of liver metastasis (arrows) (**d**). CEUS in pulse inversion mode shows a heterogeneous detection (arrows) in a later phase (**e**).

Next, we discuss the pulse inversion (PI) method and the amplitude modulation (AM) method, which are two representative modes that are frequently used in CEUS examinations [33]. Since there are some differences between the CEUS images obtained using these two methods, it is necessary to distinguish between them when performing CEUS examinations. As shown in Figure 2, the PI method is based on the sum of the first pulse and its inverted second pulse. If the tissue does not move between these two pulses and only the signal from a bubble remains unstable, then only the signal from the bubble is displayed with no background information (Figure 2a,b). In practice, however, the background signal is often not fully eliminated because of slight tissue movement. The AM method attempts to alleviate this problem of a “sooty” background. In the AM method, instead of two inverted pulses of the same magnitude (1-1), three pulses of the form 1/2-1+1/2 are emitted and summed to ensure the elimination of the signal from the background (Figure 2). Actual clinical images are presented. As shown in Figure 2, the AM method improves diagnostic accuracy by eliminating high-echo areas in the background. However, it also has the disadvantage that the image’s real-time nature is reduced as the number of US pulses increases. These two modes are available in most of the current high-end machines, but the current guidelines do not illustrate their proper use [34,35]. This increases flexibility for diagnosticians but also causes difficulties in choosing the right CEUS mode, resulting in randomness in mode selection. The basic strategy is to use one mode after another for comparison. However, the most simple and reliable method is to use the AM mode when the target lesion is highly echogenic (Figure 2c) and to otherwise use the PI mode. Nevertheless, individual adaptation should be determined each time to balance the optimal CEUS image quality, namely the maximal signal separation between the tissue and contrast medium and the optimal frame rate. However, the frame rate is crucial for accurately recording the beginning of the wash-in and wash-out. A frame rate of 10 frames/s or more is usually considered for liver tumor characterization [33].

## 3. CEUS Artifacts: How to Classify and Recognize Them

US artifacts are not restricted to B-mode US [36] but are present in all US technologies, including Doppler US [37,38] and US-derived elastography [39,40]. The use of CEUS does not allow this problem to be avoided, and a range of new CEUS-related artifacts have been reported in recent years [28,41]. Artifacts in CEUS can be divided into two groups: (1) B-mode US-related artifacts, which form the background of the CEUS image, and (2) artifacts specifically related to the CEUS method. The former includes refraction, reflection, reverberation (multiple reflections), attenuation, mirror image, and range-ambiguity artifacts [42,43]. The mechanisms of appearance of these phenomena are frequently reported as pitfalls in US diagnosis [28,43]. In the former case, the knowledge of B-mode US is sufficient for reading the displayed artifactual images. In the latter case, CEUS-specific artifacts require an understanding of the mechanism of their appearance in the CEUS image for correct image interpretation.

### 3.1. B-Mode US-Related Artifacts

B-mode artifacts represent any structure appearing in a B-mode US image that is not present in the actual tissue. The diagnostic problem is that these artifacts generate many unexpected CEUS images. However, sufficient recognition of B-mode artifacts prevents the misinterpretation of CEUS images. Representative B-mode US-related artifacts include refraction, attenuation, and range-ambiguity artifacts, as described below.

#### 3.1.1. Refraction Artifacts

**Figure 3:**
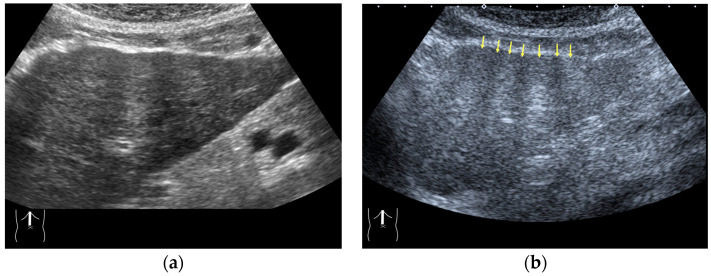
Refraction artifact (1): many vertical bands of different echogenecities in macronodular cirrhosis. (**a**) Gray-scale US reveals that the left hepatic lobe is markedly deformed with an irregular surface. The liver gives the appearance of a tricolor flag. (**b**) CEUS shows the liver to be composed of many vertical bands of different brightness (arrows), giving the appearance of a tricolor flag.

**Figure 4:**
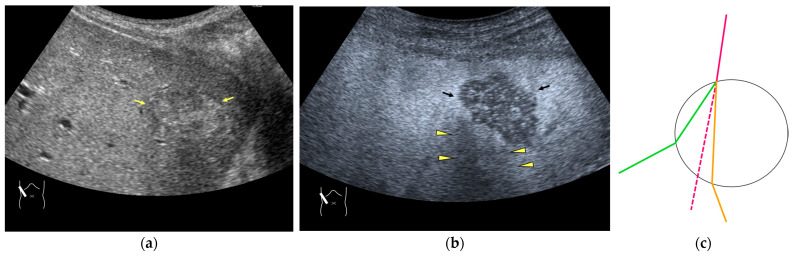
Refraction artifact (2): heterogeneous enhancement behind a mass lesion (liver metastasis). (**a**) Gray-scale US shows a 5 × 4 cm liver mass (arrows) in the right lobe. (**b**) CEUS shows it to be enhanced (black arrows, mass lesion). The liver parenchyma behind it is also coarsely enhanced (arrowheads). (**c**) A schematic drawing of sound refraction through a round mass (black circle) shows that the US beam is refracted twice at the liver parenchyma–mass lesion interface. Non-refractive lines are marked with solid and dashed pink lines. When the acoustic velocity in the mass is less than that in surrounding tissue, it is indicated with an orange line. When it is greater than that in surrounding tissue, it is indicated with a green line.

**Figure 5:**
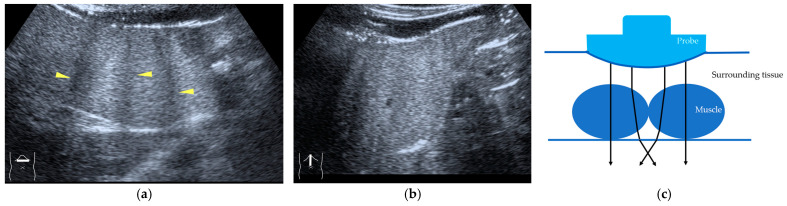
Refraction artifact (3): hypoenhanced lines due to US refraction. (**a**) CEUS shows many hypoechoic lines throughout the liver parenchyma via transverse scanning (arrowheads). (**b**) The liver’s left lobe shows none of the lines seen in (**a**) via sagittal scanning. (**c**) A schematic drawing of sound refraction due to rectus abdominus via transverse scanning plane. The US beam changes direction twice, first at the surrounding tissue–rectus muscle interface, then at the rectus muscle–surrounding tissue interface.

Current US machines reconstruct B-mode US images based on the assumption that sound passes through all parts of human tissues in a straight line and at a constant acoustic velocity (1540 m/s), and this assumption is applied to all scanning planes. The displayed position in a US image usually corresponds with the actual position on the structure. Strictly speaking, however, the acoustic velocity changes according to the tissues [44,45]. Thus, when a plane containing tissues with different acoustic velocities is scanned, sound refraction occurs at the interface between these tissues according to Snell’s law. As a result, the displayed position of point A (the true location) along the line that passes through the interface is falsely displayed at point A’ (a different position) in the B-mode US image as if there was no sound refraction. This refraction artifact is clearly seen in a cirrhotic liver (Figure 3), around a round mass (e.g., a hepatic cyst) (Figure 4) and below the rectus muscles in the transverse scanning plane of the upper abdomen (Figure 5). We will now provide a brief explanation for these three artifacts (Figure 3, Figure 4 and Figure 5). In macronodular liver cirrhosis, sound refraction occurs at the irregular hepatic surface, resulting in the improper positioning and display of echo brightness in the US image [44,45,46], giving the appearance of a “tricolor flag” [46] (Figure 3). When a US beam passes through a mass with an acoustic velocity different from that of the surrounding hepatic parenchyma, it changes direction twice due to sound refraction, first at the liver parenchyma–mass lesion entrance interface and again at the mass lesion–liver parenchyma exit interface. The liver structure behind the mass lesion thus appears to be deformed in B-mode US and heterogeneous in CEUS (Figure 4). As has been reported, sound refraction occurs most clearly at both edges of a mass lesion, and the degree of sound refraction is nearly proportional to the incidental angle of the US beam striking the liver parenchyma–mass lesion interface. Globally speaking, the degree of sound refraction is accentuated as the US beam strikes peripheral to the mass lesion. This is why the posterior echo behind the mass lesion is not homogeneous, as observed in Figure 4. In the transverse scanning of the upper abdomen, the US beam is largely refracted, first at the anterior wall of the rectus muscles and then at the posterior wall of the muscle (Figure 5c). As a result, the liver below the rectus muscles is more or less deformed in B-mode US and CEUS, as seen in Figure 5a,b. The most useful prevention strategy is to use the reference B-mode image (the so-called dual-image technique), which allows for a simultaneous comparison between the CEUS and B-mode images.

#### 3.1.2. Attenuation Artifacts

**Figure 6:**
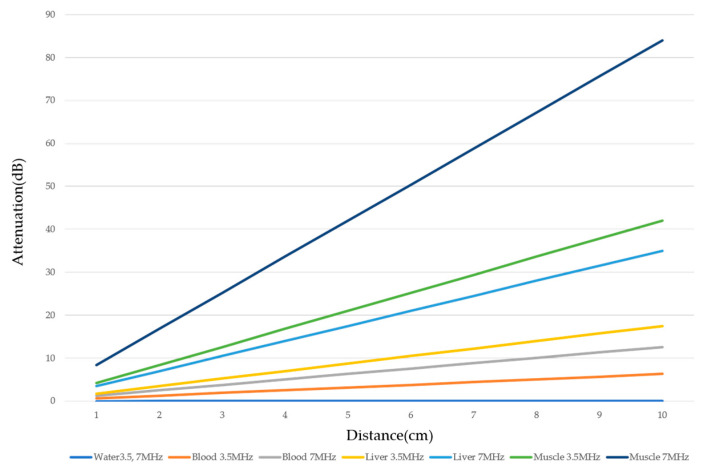
Ultrasound attenuation in relation to frequency. The US attenuation is approximately proportional to the frequency. The degree of US attenuation differs between organs. Here, we compare the degree of US attenuation of several tissues (water, blood, liver, and muscle).

**Figure 7:**
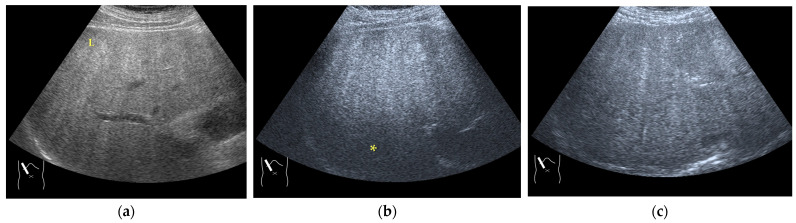
Hypoenhanced area due to sound attenuation. (**a**) Gray-scale US of the liver (L) in a patient with alcoholic liver cirrhosis. (**b**) CEUS (4 MHz) shows that the deep area is hypoenhanced (*) compared with the upper area. (**c**) CEUS (3 MHz) shows the liver to be homogeneously enhanced.

**Figure 8:**
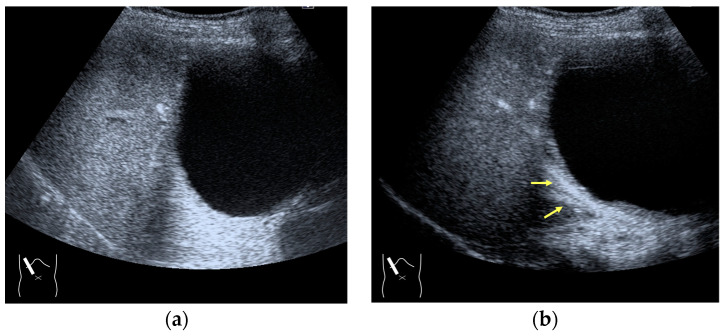
Adjustment of STC in CEUS. (**a**) Hepatic artery and portal vein are not clearly visualized before STC adjustment. (**b**) These vessels (arrows) are clearly recognizable after STC adjustment.

**Figure 9:**
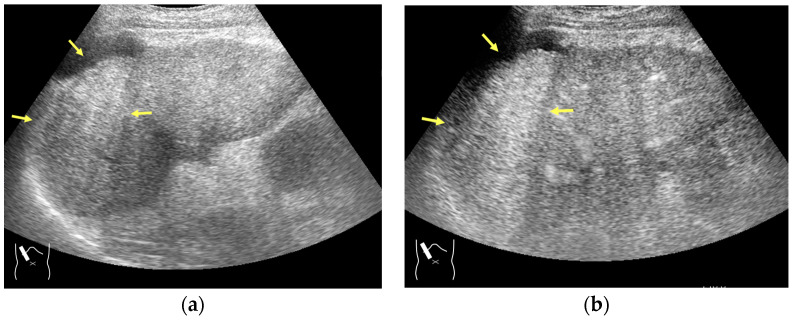
Representative case of a sound attenuation artifact. The presence of massive ascites leads to US beams passing through less attenuated ascites, mimicking an echogenic liver tumor (arrows). (**a**) B-mode US and (**b**) CEUS.

There are two sources of sound attenuation in the human body, (a) reflection and sound scattering and (b) sound absorption [47]. Both factors contribute to the formation of attenuation artifacts. These artifacts mainly occur in difficult patients (e.g., patients with advanced liver cirrhosis, patients with obesity, and patients with severe fatty liver) [48,49,50]. Increasing the mechanical index (M.I.) is not recommended for overcoming this attenuation artifact because an excessively increased M.I. causes visible microbubble destruction at the hepatic surface. Generally speaking, US attenuation in soft tissues is highly dependent on the US frequency and is nearly proportional to it (Figure 6). Less attenuation with a lower frequency results in an increased penetration depth. Thus, the most useful strategy is to use a transducer with a lower transmission frequency at the expense of a slightly deteriorated image quality (Figure 7). Another diagnostic strategy is to adjust the STC ^3)^, which slightly improves the visualization of deep areas (Figure 8). We present herein a representative case of a “pseudo-tumor” in a decompensated cirrhotic patient where less attenuated US beams passing through the ascites mimicked an echogenic tumor at the periphery of the liver not only in B-mode US but also in CEUS (Figure 9).

^3)^ STC: The most important problem in US diagnosis is the attenuation of the US beam with depth. To compensate for this attenuation, a sensitivity time control (STC) (time gain compensation (TGC)) is used to increase the amplitudes of the signals with time (depth). The greater the depth, the greater the degree of amplification [44,51,52].

#### 3.1.3. Range-Ambiguity Artifacts (RAAs)

**Figure 10:**
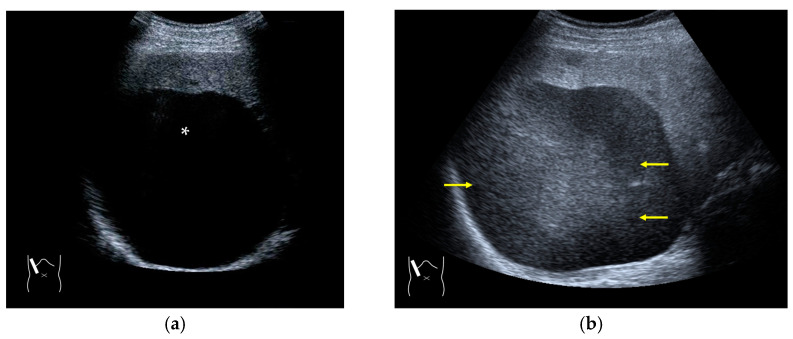

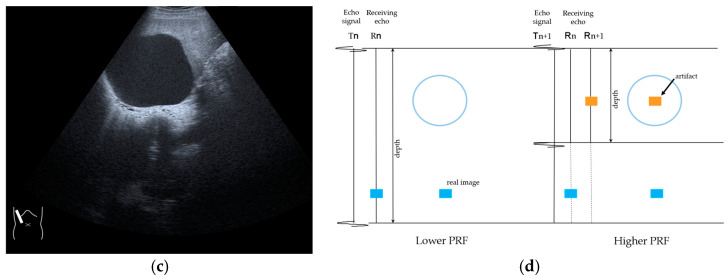
Range-ambiguity artifact in a hepatic cyst. (**a**) CEUS reveals a non-enhanced cyst (*) before the injection of the contrast medium. (**b**) Cloud-like echo (range-ambiguity artifact: RAA) appears in this hepatic cyst (arrows). (**c**) RAA disappears by changing the maximal depth of the view field. (**d**) Mechanism of RAA. When the PRF is high, the echo from the deep area is received during the second pulse’s receiving period and erroneously displayed closer to the transducer.

RAAs have recently attracted increased attention due to their increasing appearance when using recent high-end US equipment [42,43]. Although most operators encounter these artifacts in daily US examinations, they sometimes possess insufficient knowledge and feel diagnostic confusion with real structures, which is mainly because RAAs have seldom been described in the literature [42,43]. These artifacts are slightly more complex than the other B-mode artifacts. The composition of B-mode US images is based on the following assumptions: (i) the US beam passes along the same line path from the transducer to the target and back to the transducer and (ii) all received echoes come from the most recently transmitted pulse. Explaining the emission/reception of a pulse forms the basis for understanding RAAs. Assumption (ii) inevitably causes the following phenomenon: structures below the scanning depth appear in the US image when the echoes from deep structures detected with the first pulse return to the transducer after the second pulse has been emitted. The echoes coming from deep structures are, consequently, misinterpreted as having originated from the second pulse and are improperly displayed near the transducer (Figure 10). The easiest diagnostic strategy is to change the depth of the US image because this automatically changes the pulse repetition frequency (PRF) ^4)^ [42,43].

^4)^ PRF: The number of US pulses emitted per second. The pulse repetition period is the time between the beginning of a pulse’s emission and the beginning of the next pulse. In US machines, the change in the depth results in a change in the PRF [44,45].

### 3.2. CEUS-Specific Artifacts

We will now discuss CEUS-specific artifacts. These artifacts cannot be understood based on B-mode US only. They are sufficiently comprehensible via an understanding of the basic physics of CEUS. CEUS-specific artifacts include the following phenomena.

#### 3.2.1. Microbubble Destruction Artifacts

**Figure 11:**
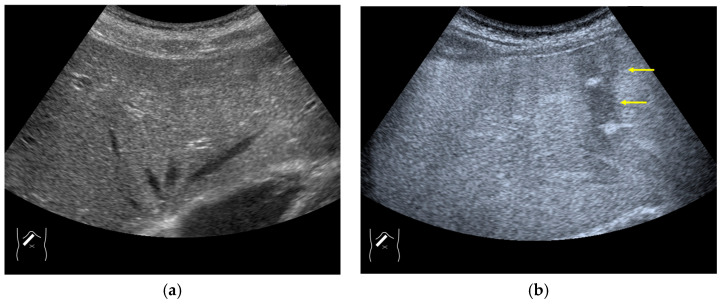
Microbubble destruction artifact. (**a**) Gray-scale US of the liver surface shows no abnormality. (**b**) Although the M.I. of the examination is not very high, a long scanning time (approximately 15 s) causes an unintentional microbubble destruction artifact (arrows) at the hepatic surface.

**Figure 12:**
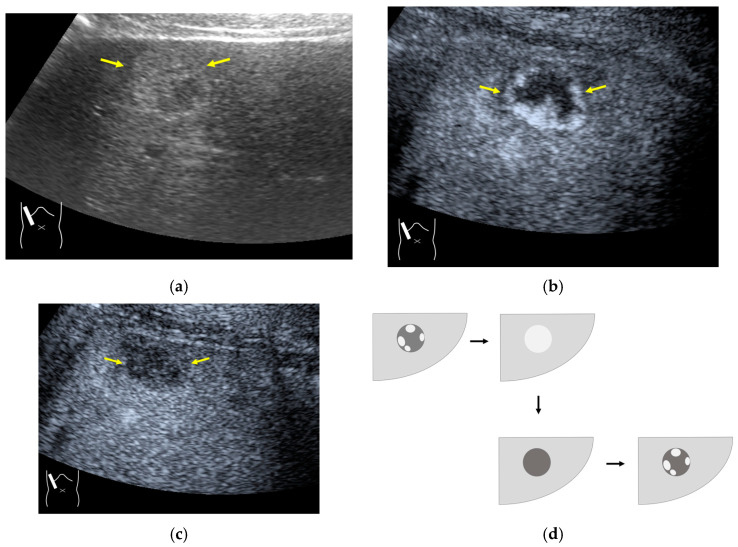
Manhole-like defect in hemangioma: (**a**) gray-scale US of the case (arrows: hemangioma); (**b**) CEUS shows a cotton wool appearance in the periphery of the lesion (arrows): (**c**) the lesion shows a complete defect in the lesion during observation (arrows); (**d**) a reasonable explanation of this phenomenon. In hemangioma, destroyed microbubbles are not quickly replaced because of the low blood flow velocity, mimicking a wash-out phenomenon.

Microbubble destruction occurs during daily CEUS examinations in all phases, even under the optimal settings. The most representative example is a microbubble destruction artifact seen at the hepatic surface (Figure 11). Thus, it is important to understand that inappropriately increased microbubble destruction occurs in the case of continuous CEUS examination, even under optimal settings. Bubble destruction artifacts cause a delicate diagnostic problem, especially when evaluating the degree of wash-out. Generally speaking, the degree of microbubble destruction differs from area to area depending on the blood flow velocity. We encounter this problem most frequently in hemangioma, where destroyed microbubbles are not quickly replaced in intrahemangioma sinusoids because of the low blood flow velocity within them [53], while destroyed microbubbles can be quickly replaced in the surrounding hepatic parenchyma (Figure 12). This phenomenon causes the important diagnostic problem of mimicking a malignant lesion. According to the CEUS LI-RADS ^5)^ classification, the presence of wash-out in the later phase suggests the diagnosis of a malignant tumor in more than 90% of cases [2,11,12,13,14,15,16]. The simplest prevention strategy is the “re-injection” of a contrast medium [54], which enables us to observe the target lesion in all phases once more, from the arterial phase until the later phase, with intermediate scanning interruptions.

^5)^ CEUS LI-RADS, like CT/MRI LI-RADS, is classified based on the probability of a lesion being HCC based on arterial phase hyperenhancement, wash-out, and other additional features, and the classification ranges between LR-1 (absolutely benign) and LR-5 (absolutely HCC). The higher the classification, the higher the probability of the lesion being HCC [1,2,11,12,13,14,15,16,29].

#### 3.2.2. Prolonged Heterogeneous Accumulation Artifacts

**Figure 13:**
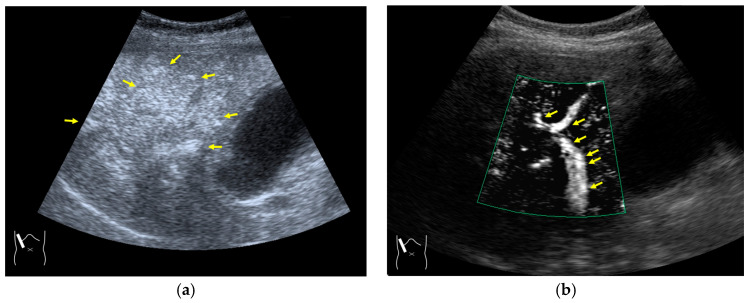
Prolonged hyperenhancement. (**a**) CEUS image in the postvascular phase. Hyperenhanced areas (arrows). (**b**) Superb microvascular imaging reveals many aggregated bubbles passing in the portal vein (arrows).

Prolonged heterogeneous liver enhancement (PHLE) is a well-known CEUS artifact [28,55], and it is characterized by the appearance of “cloudy” or “wool-like” heterogeneous enhancements in the liver’s periphery [28,55]. PHLE begins to appear within 2–6 min after contrast injection (Figure 13). Despite having no clinical significance, this phenomenon has important negative impacts on CEUS diagnosis, as it mimics focal lesions [56], intrahepatic vascular anomalies [57], or portal vein gas [58]. Although there is no established mechanism for the appearance of this phenomenon, it is assumed that, as shown in Figure 12, the contrast agent that would normally flow through the portal vein as uniform granules and uniformly reach the hepatic periphery as non-uniform granules (due to contrast agent or other causes) in the hepatic periphery, and the areas with a high concentration of the large-diameter contrast agent are expressed as heterogeneously enhanced areas. However, the precise microcirculatory and basic mechanisms leading to this phenomenon remain only partially understood, and no satisfactory conclusions have been reached. However, the occasional inhomogeneity of the diameter of the contrast agent in the portal vein is clearly recognized in recent microflow imaging (Figure 13) [59]. Although detailed proof will require many experiments and clinical cases, we hope that microflow imaging will provide a new perspective that will help to clarify these artifacts. The most useful artifact avoidance strategy is to confirm the phenomenon’s instability by changing the examination conditions.

#### 3.2.3. CEUS-Related Posterior Echo Enhancement

**Figure 14:**
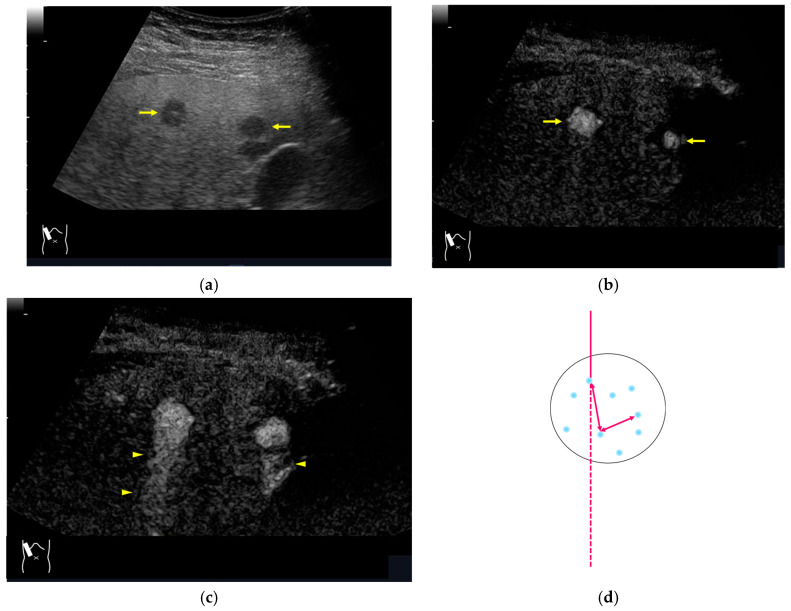
Posterior echo enhancement appearing during CEUS. (**a**) Gray-scale US of the case (arrows). (**b**) CEUS image of the lesion (focal nodular hyperplasia) (arrows). (**c**) CEUS image of posterior enhancement (arrow heads). Posterior echo enhancement appears immediately after the mass is rapidly and homogeneously enhanced. (**d**) Reasonable explanation of this phenomenon. Many scattered signals emitted from the bubbles that rapidly enter the mass lesion interfere with each other inside the stained area, and these scattered signals return to the transducer with a certain time-delay. These time-delayed signals are displayed as a PEE. Black circle: mass lesion; small blue circles: CEUS bubbles; red solid line: ultrasound beam; red dashed line: ultrasound is expected to travel; red arrows: reflection between bubbles.

CEUS-related posterior echo enhancement (PEE) differs from that of B-mode US. PEE is the most easily recognizable US artifact, and it is characterized by an echogenic band behind a lesion with sonographically different characteristics. In B-mode US, PEE is thought to be secondary to changes in the attenuation of US beams. The area distal to a less attenuating lesion exhibits an increased US intensity and is brighter than it would be without the lesion [60,61]. Posterior echoes are also considered to be strongly related to sound refraction, which occurs when the US beam strikes the interface between two media with different acoustic velocities at an oblique angle of incidence [62]. Whether the US beams converge or diverge depends on the form of the interface and whether the sound path is from a high-velocity medium to a lower-velocity medium or the opposite. The latter condition is thought to give rise to PEE. Aside from these well-known causal factors, there are many other factors contributing to PEE, including reverberation. In brief, PEE remains a multifaced entity featuring complex interplay among sound attenuation, sound refraction, sound reverberation, and other factors. In the clinical setting, hemangioma [63], hepatocellular carcinoma [61], and hepatic cysts are known to cause PEE. We sometimes encounter CEUS-related PEE in daily CEUS examinations. It is characterized by the sudden appearance of a highly echoic zone during CEUS at a location where there was no highly echoic zone in B-mode US (Figure 14). Although this phenomenon’s mechanism of appearance has not been fully elucidated, the most plausible explanation is that many scattered signals emitted from the contrast agent, which rapidly flow into the lesion, interfere with each other inside the stained area, and the reflected time-delayed signals return to the transducer with a certain delay, resulting in the appearance of a highly echoic zone behind the lesion. The precise mechanism of CEUS-related PEE is a problem to be resolved in the near future.

## 4. Conclusions

**Figure 15:**
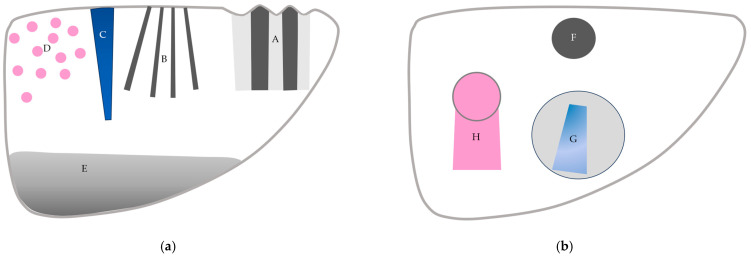
A schematic drawing of artifacts seen in CEUS of the liver. (**a**) Artifacts seen in liver parenchyma: (A,B) refraction artifacts, (C) microbubble destruction artifact, (D) prolonged heterogeneous accumulation artifact, and (E) attenuation artifact. (**b**) Artifacts seen in liver with focal lesion: (F) microbubble destruction artifact, (G) range-ambiguity artifact, and (H) posterior echo enhancement.

CEUS is used worldwide and is indispensable in diagnosing liver diseases. CEUS is extremely sensitive for detecting subtle hemodynamic abnormalities; however, it still faces many problems, including the understanding and interpretation of artifactual images. In this review, we explain the physical bases of CEUS artifacts and provide readers with representative examples in daily clinical settings. A sufficient understanding of CEUS artifacts helps to avoid the misinterpretation of CEUS images (Figure 15). Avoiding destructive artifacts due to excessively long scanning times is most important for preventing hazardous CEUS misdiagnoses.
